# Effect of budesonide oral suspension on dysphagia and esophageal inflammation in eosinophilic esophagitis: a systematic review and meta-analysis

**DOI:** 10.3389/fimmu.2026.1782316

**Published:** 2026-07-08

**Authors:** Darío S. López Delgado, Carlos A. Narváez, Gloria L. Chapues-Andrade, María A. Matus-Hernández, Veraliz González-Hidalgo, Gerson Diaz-Gonzales, Oriana Rivera-Lozada, Joshuan J. Barboza

**Affiliations:** 1 Facultad de Medicina, Universidad Cooperativa de Colombia, Pasto, Colombia; 2 Departamento de Pediatría, Hospital Escuela Cesar Amador Molina, Matagalpa, Nicaragua; 3 Facultad de Odontología, Enfermería, Medicina, Universidad Cooperativa de Colombia, Pasto, Colombia; 4 Universidad Nacional Autónoma de Nicaragua, Managua, Nicaragua; 5 Escuela de Medicina, Universidad San Ignacio de Loyola, Lima, Peru; 6 Vicerrectorado de Investigación, Universidad Señor de Sipán, Chiclayo, Peru; 7 Escuela de Medicina, Universidad Señor de Sipán, Chiclayo, Peru

**Keywords:** anti-inflammatory agents, budesonide, eosinophilic esophagitis, esophageal diseases, glucocorticoids

## Abstract

**Background:**

Eosinophilic esophagitis (EoE) is a chronic, immune-mediated esophageal disease defined by the combination of clinical symptoms of esophageal dysfunction and histological evidence of eosinophilic infiltration (≥15 eosinophils per high-power field [eos/hpf]) once secondary causes have been excluded. Tissue remodelling and impaired motility cause dysphagia and food impaction. Budesonide oral suspension (BOS) is a topical corticosteroid formulated to enhance mucosal contact and reduce inflammation. This systematic review and meta-analysis evaluated the efficacy and safety of BOS in improving dysphagia and achieving histological remission in EoE.

**Methods:**

Following the PRISMA 2020 statement, PubMed, Scopus, Web of Science, and EMBASE were searched from inception to October 14, 2024, with an update verified up to the date of submission. Randomized controlled trials (RCTs) comparing BOS (1–2 mg twice daily) versus placebo in pediatric or adult EoE patients treated for ≥12 weeks were included. The primary outcome was histologic remission (<15 eos/hpf). Secondary outcomes included endoscopic findings (Eosinophilic Esophagitis Endoscopic Reference Score, EREFS), patient-reported symptom severity (assessed with validated symptom instruments, including the Dysphagia Symptom Questionnaire, DSQ), and treatment-emergent adverse events. Random-effects meta-analyses were performed using the Paule–Mandel estimator, and certainty of evidence was graded with GRADE. Mean difference (MD; the average between-group difference in the unit of the outcome) and odds ratio (OR) were reported with 95% confidence intervals (CI).

**Results:**

Four RCTs (n = 523) met the inclusion criteria. BOS significantly improved histologic outcomes versus placebo (MD = –54.62 eos/hpf; 95% CI –68.19 to –41.05; I^2^ = 34.3%). Endoscopic severity improved (MD = –1.68; 95% CI –3.09 to –0.26). Patient-reported symptom severity also improved (pooled MD = –3.29 points; 95% CI –6.17 to –0.40), although the contributing trials used different validated symptom instruments, so this estimate reflects a composite symptom-severity effect. Treatment-emergent adverse events, mainly oropharyngeal/esophageal candidiasis, did not differ meaningfully between groups; serious adverse events were rare.

**Conclusions:**

BOS effectively reduces esophageal inflammation and alleviates dysphagia in EoE, supporting its use as a first-line topical therapy. The novel contribution of this synthesis is a strictly homogenous BOS-versus-placebo evidence base in which formulation, dose range, and follow-up are aligned across the four RCTs, complementing—rather than duplicating—broader meta-analyses that pooled heterogeneous budesonide preparations.

**Systematic Review Registration:**

https://www.crd.york.ac.uk/PROSPERO/view/CRD42025631228, identifier CRD4202525631228.

## 1 Introduction

Eosinophilic esophagitis (EoE) is a chronic, immune-mediated esophageal disease characterized by eosinophilic inflammation of the esophageal mucosa and manifesting clinically with dysphagia, food impaction and esophageal strictures (1). Incidence is approximately 5–10 cases per 100, 000 person-years and prevalence is approximately 0.5–1 case per 1, 000 (2). According to the 2018 AGREE international consensus, EoE is defined by symptoms of esophageal dysfunction together with esophageal biopsies showing at least 15 eos/hpf, after exclusion of competing causes such as gastroesophageal reflux disease, achalasia, vasculitis, hypereosinophilic syndrome, Crohn’s disease, Ehlers–Danlos syndrome, graft-versus-host disease, infection, and drug hypersensitivity (3). The diagnosis is therefore inherently clinico-histological.

Current management relies on dietary modification, proton-pump inhibitors and topical corticosteroids to control symptoms and reduce inflammation (4). Swallowed topical corticosteroids (STCs), particularly budesonide and fluticasone, are core treatments for EoE, with evidence supporting reduced eosinophilic inflammation, symptom improvement and prevention of fibrosis and dysmotility (5). Budesonide has high first-pass metabolism, minimizing systemic absorption.

Budesonide oral suspension (BOS) is a viscous corticosteroid designed to maximize esophageal contact time. In phase 3 trials, BOS demonstrated superior efficacy versus placebo, with histologic remission in about 53% of patients after 12 weeks (6). However, prior systematic reviews have pooled heterogeneous corticosteroid preparations (nebulized, orodispersible, viscous formulations, fluticasone) under the common label of “budesonide” or “topical steroids” obscuring formulation-specific signals (7–10).

Building on this background, the present review focuses exclusively on the BOS viscous formulation versus placebo in RCTs of ≥12 weeks. Compared with the most recent quantitative syntheses—including Rawla et al., (11) (12 RCTs, mixed designs, mixed budesonide formulations), Liu et al., (9) (10 RCTs, mixed budesonide formulations) and Farhan et al., (12) (11 RCTs, including orodispersible tablets and effervescent preparations)—our review narrows the PICO to a single formulation, a single dose range (1–2 mg twice daily) and the same minimum follow-up, providing a more clinically interpretable estimate for the FDA-approved BOS product. The aim is to determine the efficacy and safety of BOS in inducing histological and symptomatic remission in pediatric and adult patients, providing a focused evidence base to inform clinical practice and future head-to-head comparisons.

## 2 Methods

### 2.1 Study design

We conducted a systematic review and meta-analysis adhering to PRISMA 2020. The protocol was prospectively registered in PROSPERO (CRD4202525631228).

#### 2.1.1 Search strategy

A comprehensive search was performed across PubMed, Scopus, Web of Science, and EMBASE from inception to October 14, 2024. A controlled vocabulary (MeSH/Emtree) and free-text combination targeting “Eosinophilic Esophagitis” and “Budesonide” was used (**Supplementary Table 1**). No language or date restrictions were applied. Reference lists of included studies and relevant reviews were screened manually. The search was last verified on the date of submission, and no additional eligible RCTs published between October 14, 2024 and the submission date were identified. The verification date and the rationale for not re-running the full automated strategy are disclosed here; this limitation is discussed in section 4.6.

#### 2.1.2 Eligibility criteria

Inclusion criteria: (1) randomized controlled trials; (2) pediatric or adult patients with EoE diagnosed by clinico-histological criteria; (3) BOS (viscous formulation) 1–2 mg twice daily; (4) placebo comparator; (5) treatment ≥12 weeks; (6) PPI as background therapy when reported. Exclusion criteria: conference abstracts, non-randomized designs, case series, letters and narrative reviews. Open-label extension studies and secondary/*post-hoc* analyses of already included RCTs were excluded to avoid double counting (see **Table 1**, **Supplementary Table 2**).

**Table 1:** List of studies excluded at full-text assessment.

Study ID	Reason for exclusion
Numan et al. (8)	Wrong Study Design (Systematic review and meta-analysis, not a primary RCT).
Straumann et al. (13)	Wrong Formulation (Nebulized/swallowed, not viscous suspension).
Lucendo et al. (14)	Wrong Intervention (Orodispersible tablets, not viscous suspension).
Alexander et al. (15)	Wrong Intervention (Fluticasone vs Placebo).
Dellon et al. (16)	Wrong Study Design (Open-label extension study).

#### 2.1.3 Outcomes

The primary outcome was histologic remission, defined as a reduction of peak eosinophil counts to <15 eos/hpf (17). Secondary outcomes were: (i) endoscopic improvement (EREFS or equivalent); (ii) patient-reported symptom severity, captured with validated symptom instruments (including the DSQ); and (iii) safety (any treatment-emergent and serious adverse events, graded according to CTCAE v5.0).

#### 2.1.4 Data extraction and risk-of-bias assessment

After de-duplication, five reviewers independently screened titles/abstracts in Rayyan, followed by full-text assessment. Discrepancies were resolved by a sixth senior reviewer. Data were extracted into a standardized form, prioritizing post-treatment means and standard deviations and, when available, mean change from baseline. Where both were reported, post-treatment values were used as the primary input and change-from-baseline as a sensitivity analysis for Dellon (18) (see section 3.7). Risk of bias (RoB) was independently assessed using the Cochrane RoB 2.0 tool, classifying studies as low risk, some concerns, or high risk. RoB 2.0 judgments were re-evaluated, with particular attention to Dellon (18) (see section 3.8).

### 2.2 Statistical analysis

Random-effects meta-analyses were performed using the Paule–Mandel estimator for τ^2^ as the primary approach. Continuous outcomes were expressed as mean differences (MD = pooled between-group difference in the units of each outcome) and dichotomous outcomes as odds ratios (OR), with 95% confidence intervals (CI). Null events were handled with a continuity correction where applicable. Heterogeneity was quantified with I^2^ and τ^2^, and 95% prediction intervals were calculated for all pooled estimates.

Sensitivity analyses included: (i) re-running models with a fixed-effect approach; (ii) for histologic and endoscopic outcomes, repeating the random-effects meta-analyses with the DerSimonian–Laird estimator; (iii) for endoscopic severity, performing leave-one-out analyses, including a pre-specified analysis omitting Hirano et al. (higher overall risk of bias); and (iv) for the histologic outcome, re-running the analysis using change-from-baseline values reported by Dellon (18) (**Table 1** of the source) to verify robustness. Analyses were performed in R (v3.5.1) with the meta package. Certainty of evidence per outcome was assessed with GRADE.

### 2.3 Risk of bias and certainty of evidence

RoB 2.0 covered randomization, deviations from intended interventions, missing outcome data, measurement of outcomes, and selection of the reported result. GRADE was used to grade certainty as high, moderate, low or very low.

## 3 Results

### 3.1 Selection of studies

**Figure 1:**
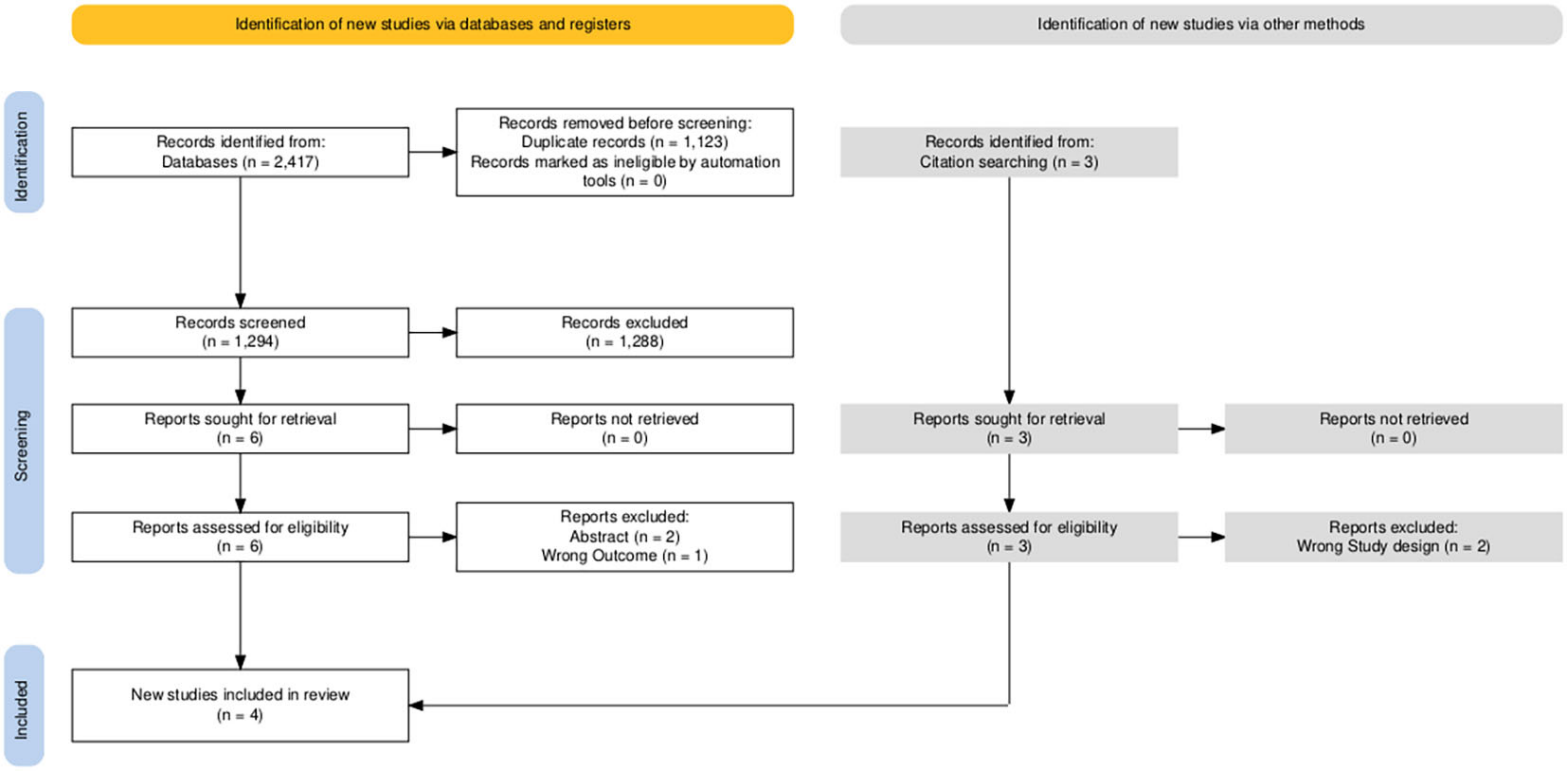
PRISMA 2020 flow diagram of study selection. Flowchart depicting study identification, screening, eligibility assessment, and inclusion. From 2, 417 records identified via databases and 3 additional records from citation searching, 1, 123 duplicates were removed, 1, 294 records were screened, 6 full-text articles were assessed for eligibility, and 4 RCTs met inclusion criteria.

A total of 2, 417 records were identified across four databases; three additional records were obtained via citation searching. After removing 1, 123 duplicates, 1, 294 records were screened. Six full-text articles were assessed for eligibility from database searching and three from citation searching; five were excluded (**Table 1**). Four RCTs (19–22) met inclusion criteria (**Figure 1**).

### 3.2 Characteristics of included studies

The four included RCTs were conducted in the United States. Three were multicenter trials [Dellon (18), Hirano (22), Gupta (21)] and one was a single-center trial Dohil (20). All used a 12-week follow-up. Detailed baseline characteristics are presented in **Table 2**.

**Table 2:** Baseline demographic and clinical characteristics of included studies.

Characteristic	Dellon et al. (2017)	Gupta et al. (2014)	Hirano et al. (2021)	Dohil et al. (2010)
Study Design	**Multicenter, randomized, placebo-controlled trial**	**Randomized, double-blind, placebo-controlled, dose-ranging trial**	**Phase 3 multicenter randomized, double-blind, placebo-controlled trial**	**Randomized, placebo-controlled trial**
Sample Size (n)	**Total: 93** **(BOS: 51, Placebo: 42)**	Total: 81* **(Analyzed: BOS 19, Placebo 21)**	**Total: 318** **(BOS: 213, Placebo: 105)**	**Total: 31** **(BOS: 21, Placebo: 11)**
Age	**Mean: 22.3 years** **(Range: 11–40)**	**Mean: 9.1 years** **(Range: 1–18)**	**Mean: 33.9 years** **(Range: 11–55)**	**Mean: 7.8 years** **(Range: 1–17)**
Gender (% Male)	**69%**	**81.5%**	**60.1%**	**84%**
Budesonide Dose	2 mg twice daily (4 mg/day)	Varied by age (0.35–4 mg daily)	2 mg twice daily (4 mg/day)	1 mg (<5ft) or 2 mg (≥5ft) daily
Treatment Duration	12 weeks	12 weeks	12 weeks	3 months
Baseline Eosinophils	Mean ~156 eos/hpf	Mean ~117.8 eos/hpf	Inclusion >15 eos/hpf	Mean ~66.7 (BOS)/83.9 (Placebo) eos/hpf

Bold values indicate the total sample size and the corresponding number of participants allocated to the budesonide oral suspension (BOS) and placebo groups, as reported in each included study.

Sample sizes varied substantially across trials, ranging from 31 to 318 participants. Two studies enrolled pediatric populations (Dohil (20): mean age 7.8 years; Gupta (21): mean age 9.1 years), one enrolled mixed adolescents/adults (Dellon (18): mean age 22.3 years; range 11–40) and one enrolled predominantly adults (Hirano (22): mean age 33.9 years). The cumulative population thus spans pediatric, adolescent and adult patients, allowing exploration of BOS effects across age strata; however, the small number of trials precluded formal subgroup analyses by age. Male predominance was observed in all trials (60.1%–84%). The intervention was uniformly BOS in a viscous formulation, with doses tailored by age/weight (0.35–4 mg/day, twice daily). Controls received matching placebo. All studies required histologically confirmed EoE (>15 eos/hpf), as expected by the standard clinico-histological diagnostic criteria (3).

### 3.3 Histologic response

**Figure 2:**
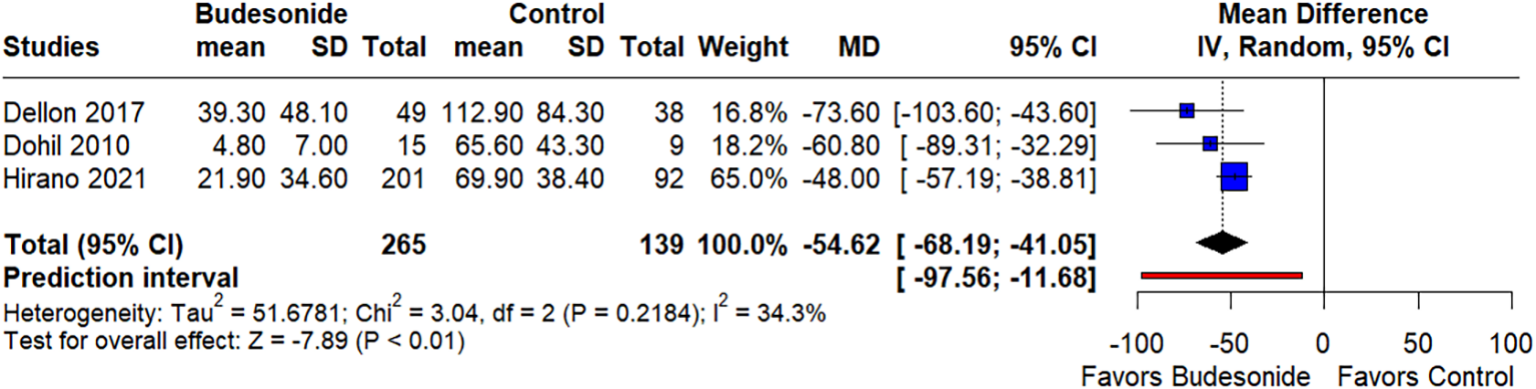
Forest plot of histologic response. Mean difference (MD) in peak esophageal eosinophil counts (eos/hpf); negative MD favors BOS. Random-effects model with Paule–Mandel (PM) estimator, 95% confidence interval (CI) and prediction interval (PI).

Across three RCTs including 404 participants (BOS n = 265; placebo n = 139), BOS produced a large reduction in peak eosinophil counts versus placebo. Using a random-effects model with the Paule–Mandel estimator on post-treatment values, the pooled MD was –54.62 eos/hpf (95% CI –68.19 to –41.05; I^2^ = 34.3%) (**Figure 2**). The prediction interval (–97.56 to –11.68) remained on the side of benefit. In a pre-specified sensitivity analysis using change-from-baseline values reported by Dellon (18) (–117.0 ± 111.6 vs –17.3 ± 83.8; **Table 1** of the source), the pooled effect direction and statistical significance were preserved (section 3.7).

### 3.4 Endoscopic severity

**Figure 3:**
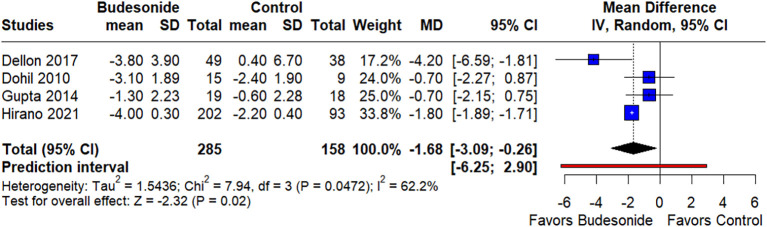
Forest plot of endoscopic severity. Mean difference (MD) in endoscopic reference scores (Eosinophilic Esophagitis Endoscopic Reference Score, EREFS, or equivalent); negative MD favors BOS over placebo. Random-effects model with the Paule–Mandel (PM) estimator, 95% confidence interval (CI) and prediction interval (PI).

Four RCTs (BOS n = 285; placebo n = 158) provided endoscopic data (EREFS or equivalent). The pooled MD was –1.68 (95% CI –3.09 to –0.26; τ^2^ = 1.54; I^2^ = 62.2%; prediction interval –6.25 to 2.90) (**Figure 3**), with consistent improvement in favor of BOS.

### 3.5 Symptom severity

**Figure 4:**
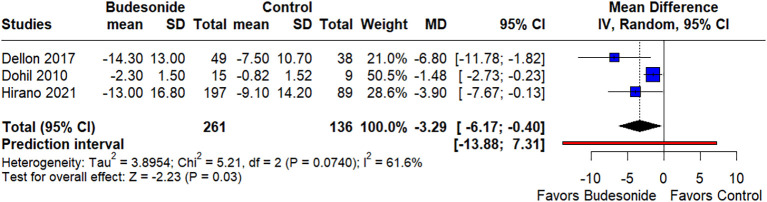
Forest plot of patient-reported symptom severity. Mean difference (MD) in symptom-severity scores; contributing trials used different validated instruments (including the Dysphagia Symptom Questionnaire, DSQ, and broader symptom-severity/total-symptom scores). Lower scores indicate less severe symptoms; negative MD favors BOS over placebo. Random-effects inverse-variance model with the Paule–Mandel (PM) estimator, 95% confidence interval (CI) and prediction interval (PI). Diamond width = 95% CI; bar = PI.

In three RCTs (BOS n = 261; placebo n = 136), the pooled MD in patient-reported symptom severity was –3.29 points (95% CI –6.17 to –0.40; τ^2^ = 3.90; I^2^ = 61.6%) favoring BOS (**Figure 4**). The prediction interval (–13.88 to 7.31) crossed the line of no effect, reflecting heterogeneity and imprecision, but the average effect indicates a clinically relevant improvement. Importantly, the contributing trials did not use a single common symptom scale: the Dysphagia Symptom Questionnaire (DSQ) was the instrument in the adolescent/adult trial, whereas the pediatric trials used broader symptom-severity scores, including a Total Symptom Score in Dohil (20) that captured esophageal symptoms beyond dysphagia (e.g., reflux-type manifestations), and a separate symptom instrument in Gupta (21) that did not employ the DSQ. Because all of these are patient-reported, oriented in the same direction (lower scores indicate less severe symptoms) and anchored on esophageal symptom burden, the pooled estimate is best interpreted as a composite symptom-severity effect rather than a pure DSQ-based dysphagia effect. We therefore pooled the trials within a random-effects model and interpret the magnitude cautiously; the instrument heterogeneity is reflected in the wide prediction interval and is acknowledged as a limitation (section 4.6).

### 3.6 Adverse events

**Figure 5:**
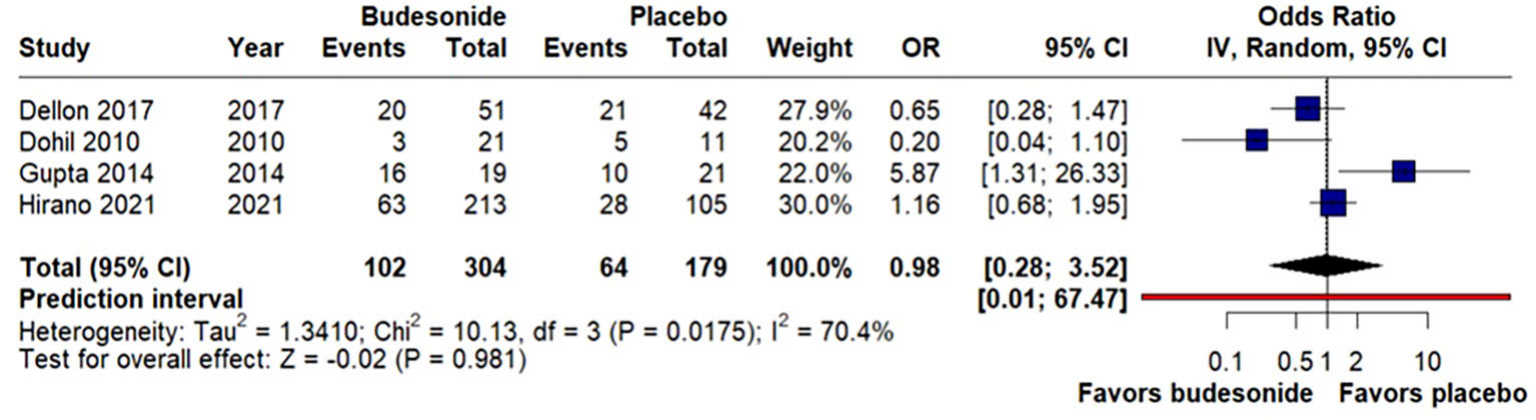
Forest plot of treatment-emergent adverse events (any grade). Odds ratios (OR); OR < 1 favors BOS. Mantel–Haenszel random-effects model, 95% CI and PI. The harmonized any-grade TEAE definition reflects that Gupta 2014 reported total adverse events without separating events by severity.

Pooled data from four RCTs (BOS n = 304; placebo n = 179) showed no clear between-group difference in the risk of any treatment-emergent adverse event (TEAE) (**Figure 5**). Because Gupta (21) reported total TEAEs without separating events by severity grade, the safety outcome was harmonized as any-grade TEAE across trials rather than mild events only; in that trial, TEAEs occurred in 13 of 21 placebo-treated participants. Safety data for Hirano were extracted from the primary Phase 3 publication (22) and its supplementary safety tables. The most frequently reported events were oropharyngeal and esophageal candidiasis. Serious adverse events were rare and did not differ between groups (3/304 with BOS vs 1/179 with placebo). Given the harmonized any-grade TEAE definition and the corrected event counts, the pooled odds ratio and its 95% confidence interval are reported in **Figure 5**.

### 3.7 Sensitivity analyses

For histologic response, results were robust. The DerSimonian–Laird estimator yielded MD –55.19 eos/hpf (95% CI –69.69 to –40.69; τ^2^ = 66.18; I^2^ = 34.3%). Using the change-from-baseline values for Dellon (18), the pooled effect remained large and statistically significant (full output in **Supplementary Material**).

For endoscopic severity, the DerSimonian–Laird model yielded MD –1.62 (95% CI –2.63 to –0.61; τ^2^ = 0.61; I^2^ = 62.2%). The leave-one-out analysis excluding Hirano (higher RoB) yielded MD –1.35 (95% CI –2.19 to –0.51; τ^2^ = 0.31; I^2^ = 50.8%; prediction interval –4.36 to 1.66), confirming that no single trial drove the pooled effect.

### 3.8 Risk-of-bias assessment

**Figure 6:**
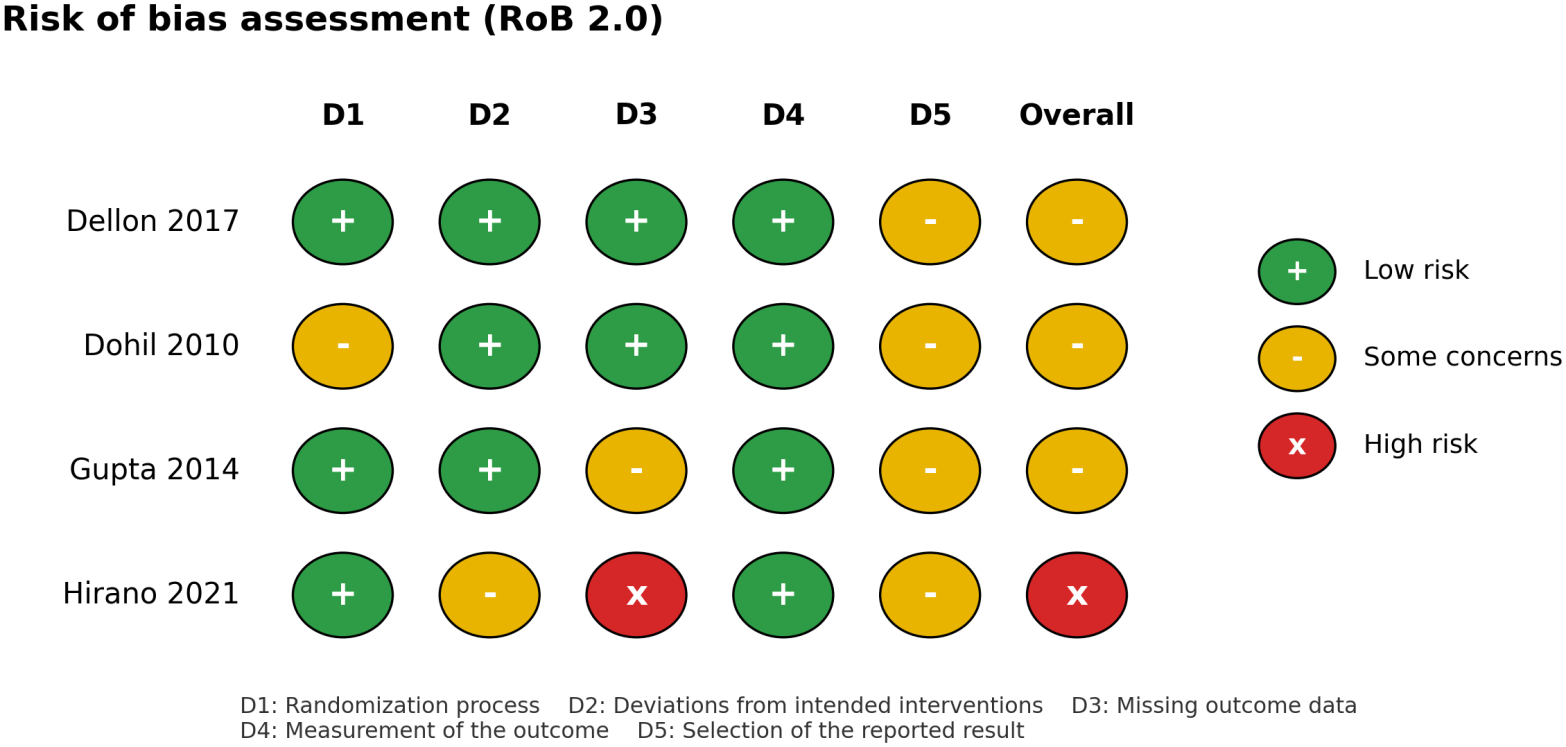
Risk of bias assessment using RoB 2.0. Domain ratings for D1 (randomization), D2 (deviations from intended interventions), D3 (missing outcome data), D4 (measurement of the outcome) and D5 (selection of the reported result). The overall judgment for Dellon 2017 is “some concerns.”.

The RoB 2.0 judgments were re-evaluated for this analysis. The updated assessment is summarized in **Figure 6** and detailed in **Supplementary Table 3**.

Specifically for Dellon (18), after re-appraisal we revised the overall judgment from “high risk” to “some concerns.” Rationale: (i) D1 (randomization) — adequate central randomization with stratification and allocation concealment described; rated “low” rather than “some concerns.” (ii) D2 (deviations from intended interventions) — the trial was double-blind and placebo-controlled, adherence was satisfactory and the analysis followed an intention-to-treat principle; we downgraded our previous “high risk” judgment to “low risk.” (iii) D4 (measurement of the outcome) — the primary endpoint (peak eosinophil count per high-power field) is objective and was assessed by blinded pathologists; we revised this domain from “some concerns” to “low risk.” (iv) D5 (selection of the reported result) was kept as “some concerns” because the trial pre-specified two co-primary endpoints (DSQ and histology) but did not provide an analytical plan locked before unblinding for all secondary endpoints. The new overall judgment for Dellon (18) is therefore “some concerns.” The remaining studies [Dohil (20), Gupta (21), Hirano (22)] retained their prior judgments.

### 3.9 Certainty of evidence (GRADE)

After updating the RoB and re-running GRADE, certainty was rated low for histologic response and serious adverse events, and very low for symptom severity, endoscopic severity and treatment-emergent adverse events (downgraded mainly for inconsistency and imprecision). Full GRADE Summary of Findings is shown in **Supplementary Table 4**.

## 4 Discussion

This systematic review and meta-analysis of four RCTs (n = 523) in pediatric and adult EoE patients confirms that BOS is superior to placebo for histologic, endoscopic and dysphagia outcomes, with a safety profile that does not differ meaningfully from placebo. Below, we explicitly position these results in the context of prior meta-analyses and articulate the specific contribution of this work.

### 4.1 Positioning relative to prior meta-analyses

Three quantitative syntheses are particularly relevant. Rawla et al., (11) (DOI 10.1007/s40268-018-0253-9) pooled 12 studies, including non-randomized designs and heterogeneous budesonide formulations (oral viscous, nebulized and effervescent preparations), reporting histologic and clinical improvement with budesonide but with high methodological variability (9, 11). Liu et al., (9) (DOI 10.1080/07853890.2022.2101689) included 10 RCTs (n ≈ 712) but combined viscous, nebulized and orodispersible formulations, reporting a standardized mean difference for eosinophil count of –1.34 (9). Most recently, Farhan et al., (12) (DOI 10.1097/MS9.0000000000004577) included 11 RCTs (n = 1, 089) and likewise mixed BOS, budesonide orodispersible tablets and effervescent budesonide, reporting an RR of 26.85 for histologic remission and an SMD of –1.08 for peak eosinophil count (12).

These syntheses convincingly support the general efficacy of “budesonide” as a drug class. They do not, however, isolate the FDA-approved viscous BOS product [EOHILIA, (23)] from other budesonide preparations, nor do they fully separate budesonide from fluticasone in pooled topical-steroid analyses. Our review intentionally restricts the PICO to: (i) viscous BOS only; (ii) a fixed dose range (1–2 mg twice daily); (iii) ≥12-week follow-up; (iv) placebo comparator; and (v) RCT designs. Consequently, our pooled estimate (MD –54.62 eos/hpf) reflects the magnitude of benefit clinicians can realistically expect when prescribing the marketed BOS product, with formulation-specific homogeneity that earlier syntheses cannot offer.

Conceptually, our work therefore complements—rather than duplicates—prior meta-analyses: (i) it provides a formulation-specific, regulatory-aligned estimate useful for guideline writers and payers comparing BOS with newer biologics (e.g., dupilumab); (ii) it incorporates updated random-effects methodology (Paule–Mandel estimator, prediction intervals, leave-one-out and alternative-estimator sensitivity analyses) that earlier syntheses did not consistently report; (iii) it reports COREOS-aligned domains (histology, endoscopy, symptoms, adverse events) that older meta-analyses treated less systematically; and (iv) it explicitly enumerates and justifies the exclusion of *post-hoc* and open-label analyses of the same trial cohort [Collins (24); Dellon (18)], which earlier syntheses occasionally double-counted. We did not extend the analysis to head-to-head comparisons with newer agents such as dupilumab, fluticasone orodispersible tablets, or anti-IL-13 monoclonal antibodies; this is acknowledged as a limitation and as a priority for future network meta-analyses (section 4.5).

### 4.2 Histologic efficacy

Hirano et al. (22) reported histologic remission in 53.1% of BOS-treated patients vs 1.0% with placebo (22). Straumann et al. (2010) reported reductions from 68.2 to 5.5 eos/hpf after 15 days of nebulized budesonide (13); Dellon et al. (25), open-label extension) reported sustained remission in nearly half of participants at 24 weeks (16). Lucendo et al. (orodispersible budesonide) reported 57.6% remission (14). Our pooled MD is consistent with these signals while being restricted to the viscous BOS formulation. Differences in formulation viscosity and mucosal contact time complicate cross-formulation comparisons (10).

### 4.3 Symptom control and dysphagia

BOS produced clinically relevant improvement in dysphagia scores. Hirano et al. reported a 52.6% response rate vs 39.1% with placebo (22). In children, symptom improvement may not parallel histologic response (18, 26), and fluticasone shows less consistent symptomatic improvement than viscous BOS or orodispersible budesonide (27). The viscous BOS formulation is particularly advantageous in pediatric populations, where the swallowing technique required for fluticasone inhalers limits its practicality, consistent with Dohil et al. (20).

### 4.4 Maintenance therapy and safety

Maintaining remission remains challenging. Dellon et al. (16) reported lower relapse with BOS over 36 weeks, although relapse still occurred in up to 43.5% of patients after cessation (16). No significant increase in serious adverse events was observed in our analysis, consistent with the low systemic absorption profile reported by Walgraeve and Vanuytsel (28) and other syntheses (9, 10, 27). Long-term HPA-axis surveillance remains advisable in pediatric populations (18, 26).

### 4.5 Strengths versus the most recent meta-analysis

Compared with the most recent prior synthesis [Farhan et al., (12)], the following specific strengths distinguish the present work:

Formulation specificity: we exclusively pooled viscous BOS (the formulation now FDA-approved as EOHILIA), whereas Farhan et al. pooled viscous BOS, orodispersible tablets and effervescent preparations. Pooling distinct delivery vehicles obscures clinically important differences in mucosal contact time and dosing.
Dose homogeneity: included trials shared a 1–2 mg twice-daily dose, while Farhan et al. pooled trials with daily doses ranging from 0.25 mg to 4 mg and once-daily to twice-daily schedules, increasing dose-related heterogeneity.
Pre-specified statistical robustness: we used the Paule–Mandel τ^2^ estimator with prediction intervals, two alternative estimators (DerSimonian–Laird, fixed-effect) and leave-one-out analyses for the highest-RoB trial. Farhan et al. used a single random-effects model without prediction intervals or estimator sensitivity analyses.
Avoidance of double counting: we explicitly excluded Collins et al., (24) (*post-hoc* EoEHSS scoring re-analysis of the Phase 2 trial) and Dellon et al., (25) (open-label maintenance extension of the same cohort). Without this safeguard, the same patients can contribute twice to a pooled estimate.
COREOS alignment: outcome reporting is structured around the Core Outcome Set for Eosinophilic Esophagitis (COREOS) (29).
Transparent re-assessment of RoB: each domain judgment is justified narratively, including the revised Dellon (19) overall rating.

The trade-off is a smaller number of trials and a narrower clinical question. We argue that this narrower question is more decision-relevant for the specific BOS product now available in clinical practice, and that our results should be read alongside—not in place of—broader syntheses (9, 11, 12).

### 4.6 Limitations and unmet needs

The small number of eligible trials precluded subgroup analyses by age and concomitant PPI therapy and limited the assessment of publication bias. The search was completed on October 14, 2024; a date-specific update was performed at submission, and no additional eligible BOS-versus-placebo RCTs were identified, but completion of pending studies (NCT05214599 and related programs) may modify these estimates. This 2024 cut-off is acknowledged as a limitation; we partially mitigated it by verifying that no additional BOS RCTs were published between October 2024 and the date of submission. The symptom outcome was measured with different validated instruments across trials (the DSQ in the adolescent/adult trial versus broader symptom-severity and total-symptom scores in the pediatric trials, the latter capturing esophageal symptoms beyond dysphagia); this heterogeneity limited the precision and comparability of the pooled estimate, which should therefore be read as a composite symptom-severity effect rather than a pure dysphagia effect. We did not perform head-to-head comparisons with biologic agents (e.g., dupilumab), which is a priority for future network meta-analyses. Most trials originated in high-income U.S. centers, which constrains external validity to populations underrepresented in current EoE research.

### 4.7 Clinical implications

BOS reliably achieves histologic remission and improves dysphagia in EoE patients, with a low rate of serious adverse events. Together with the explicit comparison against newer formulations and biologic therapies, these findings can support guideline updates and reimbursement decisions. Long-term comparative effectiveness studies remain a research priority.

In interpreting these findings, it is important to note that adverse events were harmonized according to standardized toxicity terminology, using CTCAE v5.0 as the reference framework (30), and that risk of bias was assessed using the revised Cochrane RoB 2 tool for randomized trials (31). Our findings should also be interpreted in the context of prior evidence from broader topical corticosteroid meta-analyses in eosinophilic esophagitis, which have combined different corticosteroid preparations and therefore provide less formulation-specific estimates than the present BOS-focused synthesis (32).

## 5 Conclusion

BOS is superior to placebo in inducing histologic remission and alleviating dysphagia in EoE patients. Certainty of evidence is limited by RoB in included studies, between-study heterogeneity for symptom outcomes and sparse data for serious adverse events. Within these limits, BOS is a reasonable first-line topical therapy. Higher-quality multicenter trials adhering to the COREOS outcome set and direct comparisons with newer agents (e.g., dupilumab, orodispersible budesonide, fluticasone orodispersible tablets) are necessary to define its position relative to emerging therapies.

## Data Availability

The original contributions presented in the study are included in the article/**Supplementary Material**. Further inquiries can be directed to the corresponding author.
